# Discrimination of fearful and angry emotional voices in sleeping human neonates: a study of the mismatch brain responses

**DOI:** 10.3389/fnbeh.2014.00422

**Published:** 2014-12-04

**Authors:** Dandan Zhang, Yunzhe Liu, Xinlin Hou, Guoyu Sun, Yawei Cheng, Yuejia Luo

**Affiliations:** ^1^Institute of Affective and Social Neuroscience, Shenzhen UniversityShenzhen, China; ^2^State Key Laboratory of Cognitive Neuroscience and Learning, Beijing Normal UniversityBeijing, China; ^3^Department of Pediatrics, Peking University First HospitalBeijing, China; ^4^Institute of Neuroscience, Yang-Ming UniversityTaipei, Taiwan; ^5^Department of Rehabilitation, Yang-Ming University HospitalIlan, Taiwan

**Keywords:** mismatch response, threat-related, neonate, emotional voice, anger, fear

## Abstract

Appropriate processing of human voices with different threat-related emotions is of evolutionarily adaptive value for the survival of individuals. Nevertheless, it is still not clear whether the sensitivity to threat-related information is present at birth. Using an odd-ball paradigm, the current study investigated the neural correlates underlying automatic processing of emotional voices of fear and anger in sleeping neonates. Event-related potential data showed that the fronto-central scalp distribution of the neonatal brain could discriminate fearful voices from angry voices; the mismatch response (MMR) was larger in response to the deviant stimuli of anger, compared with the standard stimuli of fear. Furthermore, this fear–anger MMR discrimination was observed only when neonates were in active sleep state. Although the neonates' sensitivity to threat-related voices is not likely associated with a conceptual understanding of fearful and angry emotions, this special discrimination in early life may provide a foundation for later emotion and social cognition development.

## Introduction

Evolution has tailored the human brain to be sensitive to the emotional expressions of others, especially when these expressions are vocalized (Hawk et al., 2009; Decety and Howard, 2013). The auditory system develops much earlier than the visual system in humans (Gottlieb, 1971). The neonatal auditory system shows advanced pitch processing capabilities which could represent pitch separately from other spectral sound features (Háden et al., 2009). Very early in development, human infants have rather extensive experience with vocal emotional information. Behavioral studies suggest that infants respond differentially to positive and negative vocal expressions (Walker-Andrews and Grolnick, 1983; Caron et al., 1988; Fernald, 1992; Grossmann, 2010). Neuroimaging studies indicate that infants at 3–7 months of age could process the voice and its emotions (Grossmann et al., 2005, 2010; Flom and Bahrick, 2007; Blasi et al., 2011; Lloyd-Fox et al., 2012). Although young infants are highly attentive to emotional voices (Grossmann, 2010), it is still not clear whether this is a basic characteristic of perception that is present at birth, or whether it is one that is learned gradually during development. Neonates (i.e., infants in the first 28 days after birth) are a group of young infants who are the least affected by early upbringing and education, and thus are the most appropriate subjects for answering this question. To date, however, there are very few studies investigating the behavioral performance or neural correlates of emotional voice processing in neonates [except one behavioral (Mastropieri and Turkewitz, 1999) and one event-related potential (ERP) study (Cheng et al., 2012)].

In addition, converging data from neuroscience have suggested that not all emotions are processed equally in the human brain (Lindquist et al., 2012). Immediate and appropriate processing of threat-related emotional (e.g., fearful and angry) cues in the environment is of evolutionarily adaptive value for the survival of individuals. The enhanced processing of social signals of threat may require little, if any, experience to develop. For instance, 4-month-old infants display an avoidant looking pattern in response to threat-related (i.e., angry and fearful) vs. non-threat-related (i.e., happy, sad, and neutral) faces (Hunnius et al., 2011). Moreover, Hoehl et al. (2008) found an ERP indicator of 3-month-old infants' attention, which is enhanced by an adult's eye gaze direction in combination with fearful relative to neutral expressions. Nevertheless, it has received little attention in neuroscience literatures that whether this heightened sensitivity to threat-related information is present at birth (Leppänen and Nelson, 2006, 2012). To our knowledge, there is only one ERP study tackling this question by investigating the brain sensitivity to threat- vs. nonthreat-related emotional voices in neonates: Cheng et al. (2012) studied the mismatch response (MMR) in reaction to emotional syllables “dada” in 1-to-5-day neonates, which found that fearful and angry syllables elicited stronger ERP amplitudes relative to happy and neutral syllables over the right hemisphere [note: the MMR is the infant equivalent of the mismatch negativity (MMN) in the adult brain].

Furthermore, although many behavioral and neuroimaging studies indicate that the brain works faster, more accurately and with enhanced neural activation in response to threat information (Vuilleumier, 2005; Williams, 2006), the model of threat-related process is oversimplified with the almost exclusive focus on the emotion of fear; other threat emotions, such as anger and disgust, are overlooked in the literature (Krusemark and Li, 2011, 2013). While fear indicates an intense urge to get out of the potential danger in the environment (Vaish et al., 2008), anger is often displayed with the aim of attack (Pichon et al., 2009) and disgust represents certain set of stimuli that would contaminate individuals both physically and psychologically (Oaten et al., 2009). Recent ERP studies found the discrimination between fear and disgust as early as 96 ms after stimulus onset (Krusemark and Li, 2011). Therefore, it is necessary to draw distinctions not only between threat and non-threat processing but also within the domain of threat (Vaish et al., 2008; Krusemark and Li, 2011). Fear and anger are both threat-related emotions (Vaish et al., 2008). The perception of fear and anger elicit comparable activity in the left amygdala, the temporal and the pre-frontal cortex (Pichon et al., 2009). However, the nature of the two emotions is qualitatively different (Fridlund, 1994; Whalen et al., 2001). Angry facial/vocal expression signals a threat-related consequence of social interaction or an attempt to control or change the behavior of others (Neuberg et al., 2011). When facing angry faces or hearing angry voices, an approach response is usually suggested to help individuals avoid danger (de Quervain et al., 2004). Whereas fear is often a reflexive response to danger and people typically adopt an avoidance response when they are fearful (Ewbank et al., 2009). Given the different evolutionary purposes of the two emotions, the current study aimed to investigate whether neonates discriminate fearful and angry emotional voices.

Finally, it should be noted that newborns spend most of their time sleeping (~20 h per day). Thus, to investigate the neural correlates of emotional voice processing during sleep is rather practical and feasible in neonates. However, the sleep state may be an important factor that affects the neural processing of emotional voices. In general, adults have two distinct sleep states: rapid-eye-movement sleep (REMS) and non-rapid eye movement sleep (NREMS) (Peirano et al., 2003). Accordingly, there are two sleep states in newborns: active sleep (AS) and quiet sleep (QS). Numerous studies have shown that the characteristics of brain activity between AS and QS differ greatly (Nunes et al., 1997; Paul et al., 2003, 2006). For instance, the MMR amplitudes show a tendency of attenuation during QS compared with AS in infants (Cheour et al., 2002; Hirasawa et al., 2002). Therefore, sleep states should be clearly discriminated and separately investigated during electrophysiological experiments. More importantly, adult neuroimaging studies have demonstrated that many emotion-related brain regions, such as the anterior cingulate cortex, orbito-frontal cortex, and amygdaloid complexes are more active in REMS than in awake and NREMS states (Kirov et al., 2012). It is highly possible that this asymmetry of emotional process between different sleep states may exist in young infants or even in neonates.

The current study investigated the auditory ERPs in 0-to-6-day neonates and compared the MMR measurements (1) between emotional vocal and acoustically matched control sounds, and (2) between fearful and angry vocal sounds. Our primary goal was (1) to provide electrophysiological evidences for neural foundations of early emotional, especially threat-related emotional, voice processing; (2) to explore whether human neonates discriminate different emotions within the domain of threat (fear vs. anger); and (3) to investigate whether the early sensitivity to threat-related emotional voices is affected by sleep states of neonates. We used the odd-ball paradigm in this study, since it has been proved to be more sensitive for investigating the capacity to distinguish among different types of stimuli, as compared with other paradigms (e.g., passive listening with equal frequency between stimuli) (Ferrari et al., 2010).

## Materials and methods

### Subjects

In total, 31 full-term neonates (16 boys and 15 girls; gestational age = 37–41 weeks, mean ± standard deviation = 39.2 ± 1.0 weeks) with post-natal ages that ranged from 0 to 6 days (2.6 ± 1.8 days) were included in this study. The inclusion criteria were: (1) birth weight appropriate for gestational age (3435.3 ± 390.5 g); (2) clinically asymptomatic at the time of electroencephalography (EEG) recording; (3) no sedation or medication for at least 48 h before the recording; (4) normal result of hearing screening with evoked otoacoustic emissions; (5) the Apgar scores at 1 and 5 min after birth were not lower than nine; and (6) normal neurologic follow-up to at least 6 months of age. The exclusion criteria were: (1) hypoxic-ischemic encephalopathy; (2) intraventricular hemorrhage or white matter damage observed on cranial ultrasound; (3) major congenital malformation; (4) central nervous system infection; (5) metabolic disorder; (6) clinical evidence of seizures; and (7) evidence of asphyxia. Another four neonates were recorded but were not included in the analyses because of large motion artifacts resulting in too few clean trials.

Informed consent was signed by the parent or legal guardian of the neonates to approve the use of clinical information and EEG data for scientific purpose. The research protocol was approved by the Ethics Committee of Peking University.

### Stimuli

The stimulus materials consisted of the fearfully and angrily spoken syllables “dada” and their corresponding non-vocal sounds (refer to Cheng et al., 2012 for more details). In brief, a young female speaker produced the syllables of “dada” with emotional prosodies of fear and anger (each for more than 10 times). Sounds were then rated for emotionality (five-point scale) by 120 adults. Two emotional sounds that had been consistently identified as extremely fearful and extremely angry (score = 5) by all the 120 raters were selected as the vocal stimuli. With the use of Cool Edit Pro 2.0 and Sound Forge 9.0, emotional syllables “dada” were edited to have equal duration (350 ms) and mean intensity [min: 57 dB; max: 62 dB; mean: 59 dB sound pressure level (SPL)] (see Cheng et al., 2012; Fan et al., 2013; Hung et al., 2013 for validation).

**Figure 1:**
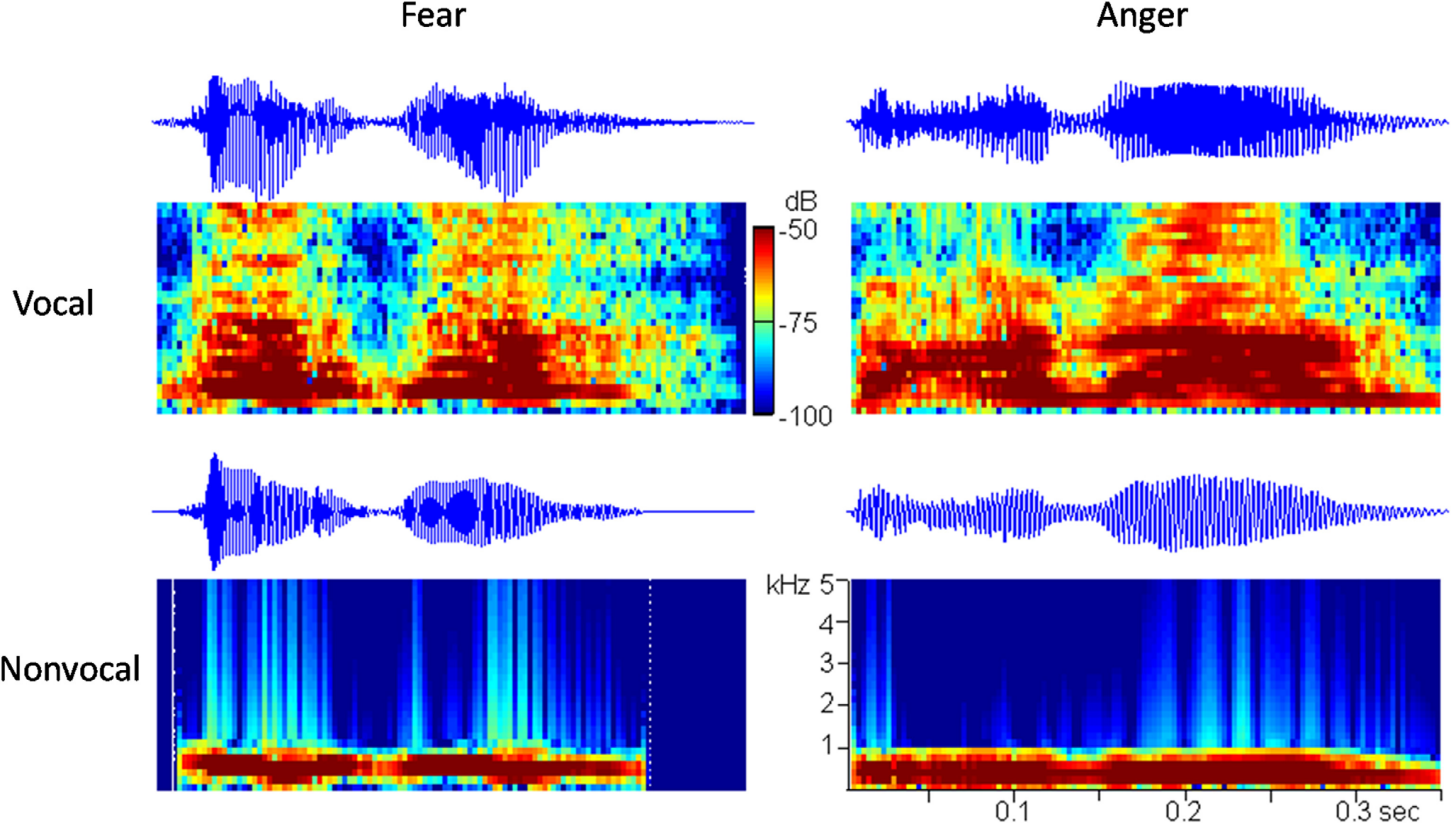
**The oscillograms and the spectrograms of the four auditory stimuli used in this study**.

It is known that prosodic characteristics of speech (i.e., frequency, intensity, and rhythm) play an essential role in the perception of vocally communicated emotions (Scherer, 1986). It is hypothesized that the low-level acoustical features (e.g., fundamental frequency) are not sufficient to provide emotional content in voices, and that newborns may rely on many prosodic cues, such as frequency, intensity, and rhythm to infer emotions. In order to exclude the possibility that newborns discriminate among emotional voices that differ with respect to their low-level acoustical features, we followed the method of Cheng et al. (2012) and used another set of control sounds in the experiment. Since fundamental frequency (f0) and intensity are the most correlative acoustical variables of emotions (Banse and Scherer, 1996), the non-vocal sounds were produced to follow the f0 contours and the temporal envelopes of the corresponding vocal sounds (i.e., non-vocal sounds preserved the temporal and spectral f0 features). The two non-vocal sounds were modified from the fearful and the angry vocal sounds using Praat (Boersma, 2002) and Matlab software (The MathWorks, Inc., USA). The oscillograms and the spectrograms of the four stimuli used in this study are shown in Figure 1.

### Procedure

The experiment was performed in the neonatal ward of Peking University First Hospital, Beijing, China, during September 2012 and February 2013. The mean background noise level (without speech material presentation) was 30 dB SPL (Brüel and Kjær, Nærum, Denmark; sound analyzer type 3160-A-042; 2cc-coupler type 4157). Sounds were presented to neonates via insert earphones (XBA-20/SQ, Sony, Tokyo, Japan).

In this study, the emotional MMR was investigated using fearful sounds as standard stimuli (80%) and angry sounds as deviant stimuli (20%). The standard and the deviant stimuli were randomly presented during the experiment. Each deviant followed at least two standards. The interstimulus interval varied randomly between 550 and 750 ms, i.e., the mean stimulus onset asynchrony was 1000 ms (Cheour et al., 2002; Hirasawa et al., 2002).

There were two kinds of blocks in the experiment. The vocal block consisted of 240 fearful and 60 angry vocal sounds; the non-vocal block consisted of 240 fearful and 60 angry non-vocal sounds. The vocal and non-vocal blocks were presented alternately during the experiment, with a 10-s silent period between adjacent blocks. Considering that neonates typically begin a sleep episode in AS and episodes of AS and QS alternate with a period of 50–60 min (Peirano et al., 2003), the sound stimuli in this study were continuously presented for approximately 1 h, containing a total of six vocal and six non-vocal blocks.

**Figure 2:**
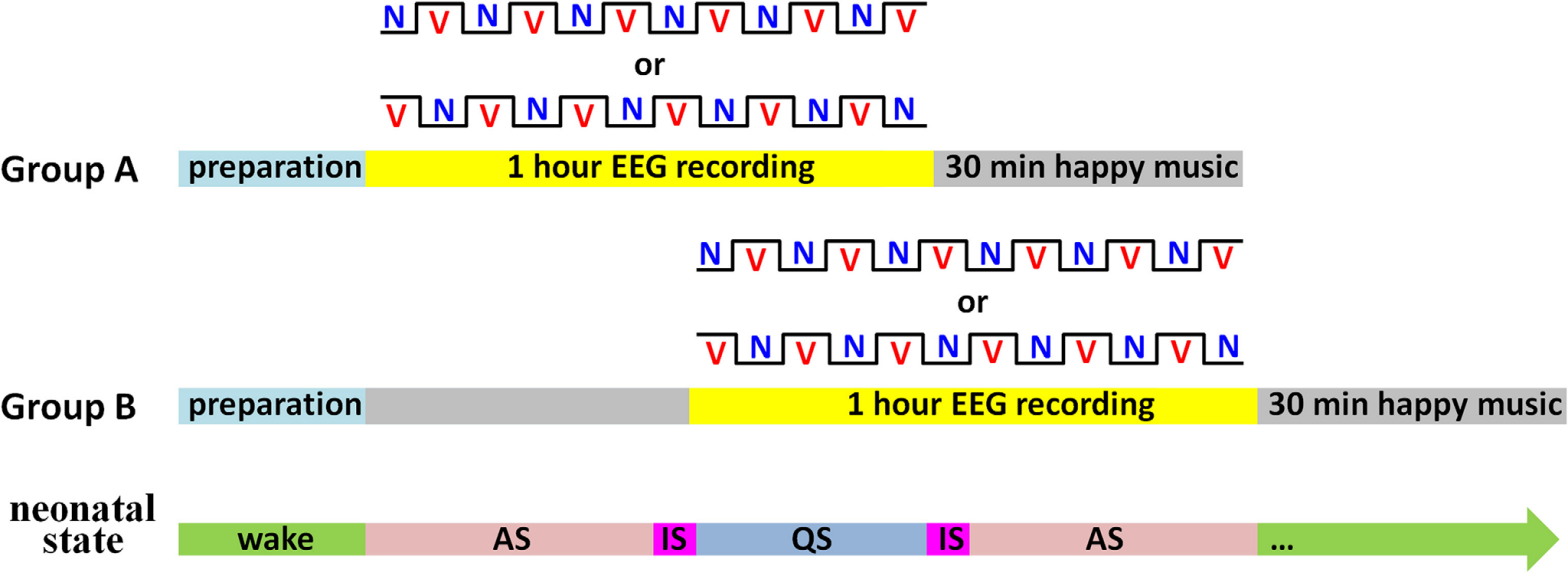
**Illustration of the experimental procedure**. There were two kinds of blocks: V for the vocal block and N for the non-vocal block. Sleep states: AS for active sleep, QS for quiet sleep, and IS for indeterminate sleep (i.e., periods between AS and QS).

During the experiment, a sleep state recorder was employed to monitor the neonatal states online. As shown in Figure 2, subjects were randomly divided into two groups. In Group A (*n* = 16), the 1-h EEG recording was initiated as soon as the neonates entered AS state. In contrast, the EEG recording in Group B (*n* = 15) was initiated when the first AS period finished. In each group, half of the neonates began the EEG recording with the vocal block and another half began with the non-vocal block.

In order to remove the potential negative mood induced by emotional “dada” sounds, *Twinkle, Twinkle Little Star Variations* (composed by Wolfgang Amadeus Mozart) and *Jingle Bells* (composed by James Lord Pierpont) were played on a continuous loop for 30 min (mean intensity = 55 dB SPL) immediately following the EEG recording.

### EEG and aEEG recording

The EEG and electrooculogram (EOG) were recorded referentially against left mastoid and off-line re-referenced to the average of the left and the right mastoids (HANDYEEG, Micromed, Treviso, Italy). Two EOG electrodes were placed above and below the right eye for vertical eye movement recording and another two electrodes were placed on the left and right external canthi for horizontal eye movement recording. Considering the fronto-central distribution of the neonatal MMR (Näätänen et al., 2007; Cheng et al., 2012), the current study investigated the EEG data at electrodes FC3, FC4, CP3, and CP4 in the international 10–20 system. The sampling frequency was 256 Hz. The electrode impedances were kept below 5 kΩ.

**Figure 3:**
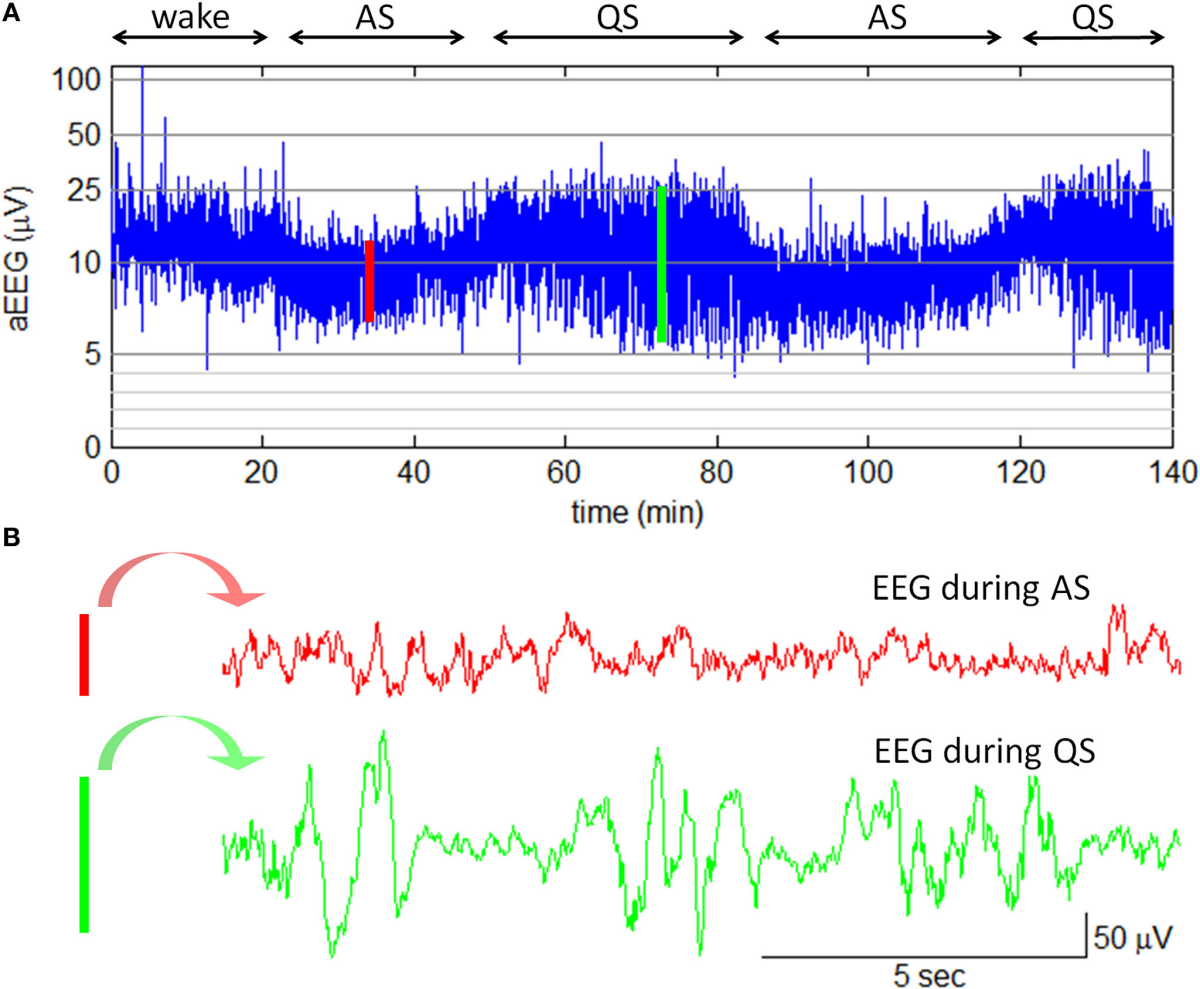
**Neonatal sleep EEG and aEEG**. **(A)** aEEG waveform over 140 min. The cyclicity of aEEG tracings in full-term neonates is characterized by smooth periodic changes in bandwidth. The broad bandwidth represents the relatively discontinuous background activity during QS while the narrow bandwidth corresponds to the more continuous activity during AS. Note: the aEEG during the wake state (the first 20 min in this plot) was contaminated with motion artifacts. **(B)** Two 15-s epochs of EEG waveform. The EEG epoch in red is corresponding to the red line in aEEG tracing; the EEG epoch in green is corresponding to the green line in aEEG tracing.

In this study, amplitude-integrated EEG (aEEG) was employed with the goal of bedside sleep state monitoring. The aEEG is a simplified EEG system that makes use of ongoing EEG amplitudes in single channel. In this system, the raw EEG signals from biparietal electrodes are amplified, filtered, and compressed over long periods of time to obtain a transformed EEG waveform that enables evaluation of long-term trends in electrocortical background activity (Hellström-Westas et al., 2006). The aEEG data were recorded with two detecting electrodes at P3 and P4 and with a ground electrode at Fz (2-channel USB, Symtop Instrument, Beijing, China). The impedance was kept below 5 kΩ during the recording. The aEEG tracings were calculated online according to the algorithm previously described by Zhang et al. (2011). In brief, the filtered EEG signals were divided into non-overlapping epochs of 15-s duration. The maximum and the minimum of peak-to-peak amplitudes in each EEG epoch were extracted as the upper and the lower terminal points of the associated aEEG vertical line (refer to Figure 3). To give a bird's-eye view of cerebral function over a long duration, data compressions both in the amplitude scale (y-axis) and in the time scale (x-axis) were performed. In amplitude compression, the aEEG vertical line was drawn with a log-scale y-axis. Time compression of the aEEG tracing was achieved with the use of a single aEEG vertical line to replace a 15-s EEG segment. During the experiment, neonatal states (wake, AS, or QS) were recorded by a pediatric neurologist based on clinical observations and aEEG tracings. The recorded states were further confirmed by an experienced neurophysiologist who had full access to simultaneous raw EEGs. An example of neonatal sleep aEEG is shown in Figure 3.

### ERP analysis and statistics

Two 20-min EEG segments were manually selected according to the sleep states revealed by aEEG tracings: one from the middle of AS period and the other from the middle of QS period. The 20-min EEG data contained 120 angry and 480 fearful vocal sounds, and 120 angry and 480 fearful non-vocal sounds. The vertical and the horizontal EOG data were transformed offline into bipolar signals. Both vertical and horizontal ocular artifacts were removed from the EEG data using a regression procedure implemented in Neuroscan software (Scan 4.3). After EOG correction, the EEG data were filtered with a 0.01–30 Hz finite impulse response filter with zero phase distortion. Filtered data were segmented beginning 200 ms prior to the onset of vocal or non-vocal sounds and lasting for 1000 ms. EEG epochs containing large artifacts (> ±100μV) were rejected. Epochs were baseline-corrected with respect to the mean voltage over the 200 ms preceding the onset of the sounds, followed by averaging in association with experimental conditions.

The auditory MMN response in adults is with fronto-central scalp distribution and is negative in polarity at about 150–250 ms post-stimulus. MMN is a pre-attentive component of the auditory ERP that shows a negative displacement in response to deviant sounds compared to standard sounds in the odd-ball paradigm (Näätänen, 1992). The MMN can be elicited in the absence of attention, which is especially promising for recording young infants (Näätänen et al., 1993). Since in some infants a positive component has been reported instead of a MMN (e.g., Dehaene-Lambertz, 2000; Friederici et al., 2002; Winkler et al., 2003; Leppänen et al., 2004; Ruusuvirta et al., 2009; Cheng et al., 2012), we named this ERP component as MMR in this study.

**Figure 4:**
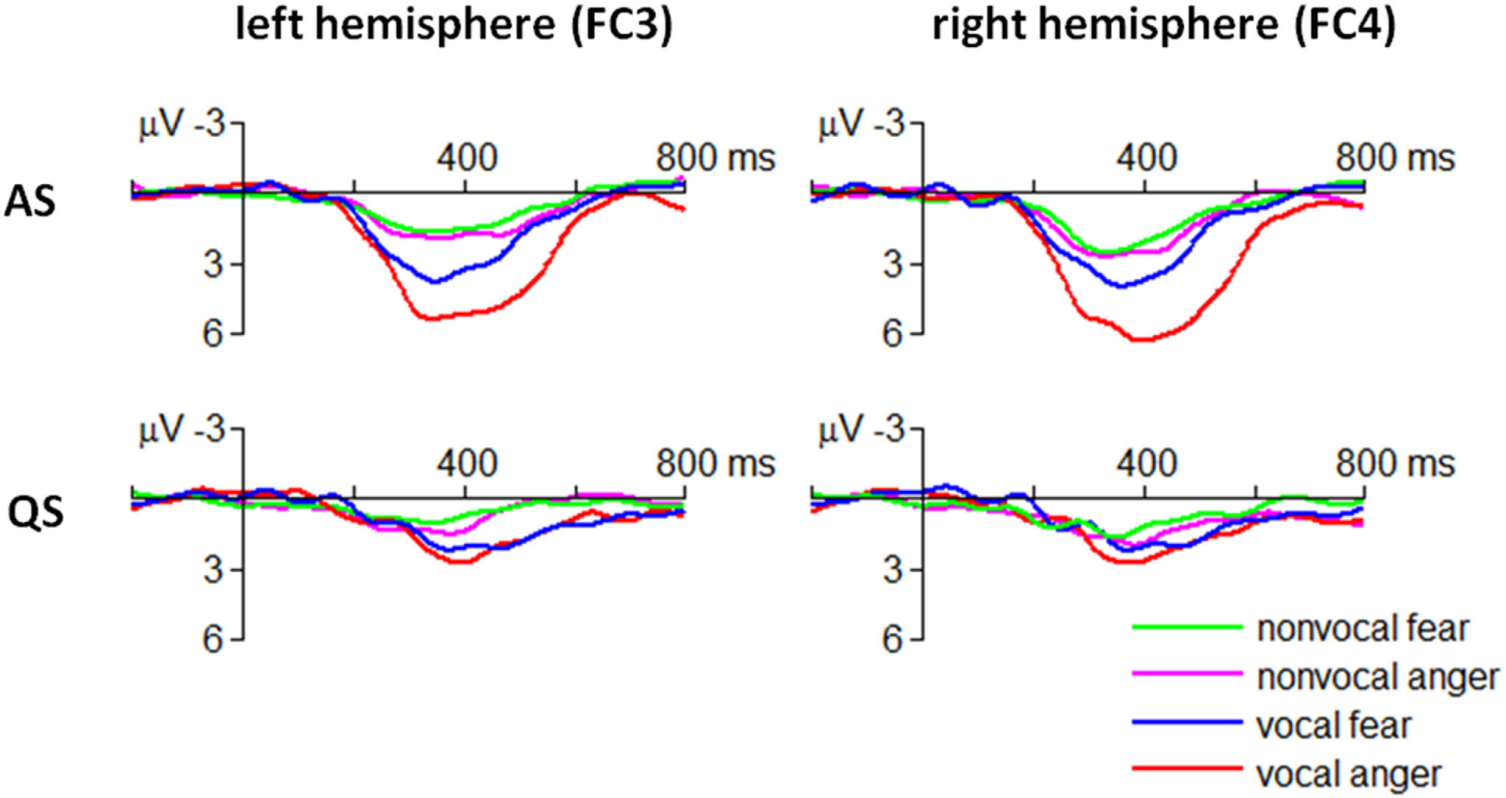
**Grand-mean ERP waveforms in fearful and angry conditions**.

Considering the latency of the MMR decreases as a function of age (Friederici et al., 2002), this study focused on the mean amplitudes of ERP within a time window of 300–500 ms post-stimulus, which was consistent with the neonatal MMR study by Cheng et al. (2012). Although the grand-mean ERPs were plotted using all the clean trials (Figure 4), only 1/4 vocal and non-vocal fearful trials (standard stimuli) were used (with random selection) to statistically compare with angry trials (deviant stimuli). The number of accepted trials per condition (for statistical analyses) was 92.3 (81–108), 89.5 (79–98), 90.7 (79–104), and 89.8 (74–102) in angry vocal, fearful vocal, angry non-vocal, and fearful non-vocal conditions, respectively (mean, range).

Statistical analyses were performed using SPSS Statistics 20.0 (IBM, Somers, USA). The significance level was set at 0.05. A Four-Way repeated measures ANOVA on the mean amplitudes of MMR component was conducted with emotion (fear/anger), sound type (vocal/non-vocal sound), sleep state (AS/QS), and electrode (FC3/FC4/CP3/CP4) as within-subjects factors. Greenhouse-Geisser correction for ANOVA tests was used whenever appropriate. *Post-hoc* testing of significant main effects was conducted using Bonferroni method. Significant interactions were analyzed using simple effects models. Partial eta-squared (η^2^*_p_*) was reported to demonstrate the effect size in ANOVA tests, where 0.05 represents a small effect, 0.10 indicates a medium effect, and 0.20 represents a large effect. For the sake of brevity, effects that did not reach significance have been omitted.

## Results

### Four-way interaction

The ANOVA revealed that the four-way interaction of emotion × sound type × sleep state × electrode was significant for the ERP amplitudes within 300–500 ms [*F*_(3, 90)_ = 4.05, *p* = 0.009, η^2^_p_ = 0.119] (Figure 4).

In particular, the MMR evoked by angry vocal sounds (FC3 = 5.09 ± 2.50 μV; FC4 = 6.06 ± 2.89 μV) was significantly larger than that evoked by fearful vocal sounds (FC3 = 3.45 ± 2.25 μV; FC4 = 3.37 ± 2.82 μV) during AS state at fronto-central electrodes [FC3: *F*_(1, 30)_ = 7.07, *p* = 0.012; FC4: *F*_(1, 30)_ = 16.6, *p* < 0.001]; this effect was not significant at centro-parietal electrodes or during QS state (*p*s > 0.05). Moreover, the result indicated that the emotional effect of vocal sounds had a slight right-hemisphere advantage.

The MMR evoked by non-vocal sounds did not show fear vs. anger difference in each combination of sleep state × electrode conditions (*p*s > 0.05).

### Two-way interaction

There were four significant two-way interactions. The emotion × sleep state interaction was significant [*F*_(1, 30)_ = 16.3, *p* < 0.001, η^2^_p_ = 0.352]. Simple effect analysis revealed that the MMR evoked by angry “dada” sounds (3.23 ± 3.18 μV) was significantly larger than that evoked by fearful “dada” sounds (1.81 ± 2.30 μV) during AS state [*F*_(1, 30)_ = 26.6, *p* < 0.001]. However, this effect was not significant during QS state (*p* > 0.05).

The emotion × electrode interaction was significant [*F*_(3, 90)_ = 9.04, *p* < 0.001, η^2^_p_ = 0.232]. Simple effect analysis revealed that the MMR evoked by angry sounds (FC3 = 3.08 ± 2.90 μV; FC4 = 3.44 ± 3.28 μV) was significantly larger than that evoked by fearful sounds (FC3 = 2.03 ± 2.55 μV; FC4 = 2.10 ± 2.69 μV) at fronto-central electrodes [FC3: *F*_(1, 30)_ = 6.77, *p* = 0.014; FC4: *F*_(1, 30)_ = 8.51, *p* = 0.007]. However, this effect was not significant at centro-parietal electrodes (*p*s > 0.05).

The sound type × electrode interaction was significant [*F*_(3, 90)_ = 9.78, *p* < 0.001, η^2^_p_ = 0.246]. Compared with non-vocal sounds (FC3 = 1.67 ± 2.62 μV; FC4 = 2.00 ± 2.66 μV), vocal sounds elicited significantly larger MMR amplitudes at fronto-central electrodes (FC3 = 3.44 ± 2.64 μV; FC4 = 3.54 ± 3.27 μV) [FC3: *F*_(1, 30)_ = 37.2, *p* < 0.001; FC4: *F*_(1, 30)_ = 21.9, *p* < 0.001]. However, this effect was not significant at centro-parietal electrodes [*F*_(1, 30)_ < 1].

The sleep state × electrode interaction was significant [*F*_(3, 90)_ = 11.9, *p* < 0.001, η^2^_p_ = 0.284]. The MMR was significantly larger during AS (FC3 = 3.35 ± 2.81 μV; FC4 = 3.84 ± 2.94 μV), than during QS state (FC3 = 1.76 ± 2.51 μV; FC4 = 1.71 ± 2.83 μV) at fronto-central electrodes [FC3: *F*_(1, 30)_ = 22.2, *p* < 0.001; FC4: *F*_(1, 30)_ = 29.9, *p* < 0.001]. However, this effect was not significant at centro-parietal electrodes (*p*s > 0.05).

### Main effect

There were three significant main effects in our data. The main effect of sound type was significant [*F*_(1, 30)_ = 35.6, *p* < 0.001, η^2^_p_ = 0.543). The MMR was larger in response to vocal (2.30 ± 2.96 μV) relative to non-vocal sounds (1.50 ± 2.51 μV).

The main effect of sleep state was significant (*F*_(1, 30)_ = 23.2, *p* < 0.001, η^2^_p_ = 0.437). The MMR was larger during AS state (2.52 ± 2.86 μV) than during QS state (1.28 ± 2.53 μV).

The main effect of electrode was significant (*F*_(3, 90)_ = 41.8, *p* < 0.001, η^2^_p_ = 0.582). The MMR was larger at fronto-central electrodes (FC3 = 2.56 ± 2.77 μV; FC4 = 2.77 ± 3.07 μV) compared to that at centro-parietal electrodes (CP3 = 1.27 ± 2.38 μV; CP4 = 1.00 ± 2.36 μV) (*p*s < 0.001).

### Results of the additional experiment

In order to clarify that the observed ERP difference between angry and fearful voices was not simply due to the stimulus frequency (20 vs. 80%), an additional experiment was performed, with a balanced design in which angry and fearful voices were given at equal probability (50 vs. 50%). The result of the additional experiment was consistent with the result of the odd-ball experiment, with larger ERP amplitudes in response to angry voices in comparison with those to fearful voices. Please refer to the Supplementary Materials for more details of the additional experiment.

## Discussion

Human voices, which convey important affective information, play a fundamental role in social communication (Cheng et al., 2012). Numerous adult studies have revealed that auditory stimuli spoken in different emotional categories are encoded by distinct ERP/fMRI patterns in the brain (Alter et al., 2003; Schirmer and Kotz, 2003; Wambacq and Jerger, 2004; Wambacq et al., 2004; Ethofer et al., 2009). However, the processing of emotional prosody at the very early days of life is still far from clearly understood (Grossmann et al., 2005). One behavioral study presented newborn babies with vocal expressions of happy, angry, sad, and neutral emotions, which found an increase in eye-opening responses following happy voices compared with the other emotional voices while neonates listened to the voices in their native language (Mastropieri and Turkewitz, 1999). To understand the neural bases of emotion processing in early development, Cheng et al. (2012) investigated, for the first time, the ERP responses to different emotional prosodies in neonates; the authors found that the MMR for affective discrimination between negative (fearful or angry) and happy voices was already present during the neonatal period. As a follow-up study of Cheng et al. (2012), the current study revisited the MMR component in response to the “dada” emotional prosodies in 0-to-6-day neonates. In general, this study had two novel findings that might deepen our understanding of early emotional voice processing.

Firstly, we found that the fronto-central scalp distribution of neonatal brain could discriminate fearful voices from angry voices. The previous two neonatal studies mainly focused on the valence-based emotional voice processing, so they could not determine whether different emotions of the same valence differentially influence the behavior and the brain activity of newborns (Mastropieri and Turkewitz, 1999; Cheng et al., 2012). The emotional voices of fear and anger are universal social signals that frequently signify threat to perceivers. While fear and anger are both threat-related emotions, the corresponding adaptive responses are quite different: compared with fear, anger is often displayed with the aim of altering the behavior and therefore appears to be a more interactive signal (Pichon et al., 2009). The brain's processes of fearful and angry information probably have different neural mechanisms. Adult fMRI studies have demonstrated that the brain activity in the amygdala displays different patterns in response to fearful vs. angry faces/actions (Whalen et al., 2001; Pichon et al., 2009); and the perception of anger triggered a wider set of regions comprising the anterior temporal lobe as compared with fear perception (Pichon et al., 2009). In line with the findings observed in adult studies, our data showed that compared with the standard fearful prosody, the ERP amplitude was larger in response to the deviant angry prosody, indicating that the neonate's brain is sensitive to different threat-related emotional voices. In addition, to verify that the ERP-based perceptual difference was driven by the prosodic cues that contained emotional content rather than only low-level acoustical properties, we employed another set of control stimuli, in which non-vocal sounds were matched for low-level acoustical structures, such as mean intensity, mean fundamental frequency, the temporal variability of intensity, and the temporal variability of fundamental frequency (see similar methods in Belin and Grosbras, 2010; Grossmann, 2010; Cheng et al., 2012). We found that the fear–anger MMR effect was present for emotional voices but absent for low-level acoustical controls. Present ERP results provide evidence that threat-related fearful and angry voices could be separately processed in neonates' brain based on their prosodic cues and that the effect does not reflect a simple response to the low-level acoustical features of vocal sounds.

Secondly, our data provide preliminary evidence that the brain's response to emotional sounds is affected by sleep state of neonates: the emotion × sleep state interaction was significant; the fear–anger MMR discrimination was observed only during AS state. This result is in agreement with Beauchemin et al. (2011), who also considered sleep state as an important factor and investigated the neonatal auditory ERPs only when the subjects were in AS state. It should be noticed that some infant/neonatal studies did not find differences in MMR between asleep and awake stages, or between AS and QS states (Hirasawa et al., 2002; Martynova et al., 2003; but see Friederici et al., 2002). The inconsistent results of the effect of different brain states on neonatal MMR may be due to two reasons. First, we used emotional voices in this study while the previous studies employed pure tones (Hirasawa et al., 2002) or vowels (Martynova et al., 2003). Second, most previous studies discriminated the neonatal state mainly based on the review of video cameras (e.g., Hirasawa et al., 2002) or EEG data (e.g., Martynova et al., 2003). This study used aEEG algorithm to online monitor the neonate's state. The aEEG method highly simplified EEG interpretation by compressing the long-term EEG data into a short compact tracing and thus potentially enhanced the accuracy of arousal level estimation (refer to Figure 3).

Consistent with Cheng et al. (2012), we found that human emotional voices enhanced the MMR amplitude at fronto-central electrodes (see also Levy et al., 2003), and with a slight tendency of right-hemisphere advantage (see also Grandjean et al., 2005; Ethofer et al., 2006; Schirmer and Kotz, 2006; Belin and Grosbras, 2010; Grossmann et al., 2010). It has been shown that the MMR may be generated by neural sources in the superior temporal sulcus (STS) (Maurer et al., 2003; Näätänen et al., 2007; Herrmann et al., 2009); and that the STS of adult's brain (especially at the right hemisphere) is consistently found in response to emotional voices (Belin et al., 2000; Binder et al., 2000; Scott et al., 2000; Wiethoff et al., 2008; Ethofer et al., 2012). Although the limited spatial resolution of ERP method prevented us from deciding whether the STS (or the right STS) is the exact neural base for emotional voice processing in neonates, the present study at least suggests a tendency of early cerebral specialization for the automatic perception of threat-related emotional voices.

In addition, this study found a positive MMR instead of a typical negative MMN in the neonatal brain. Unlike the negative difference wave to voices in adults, many studies found that the fronto-central MMR to the discrimination between different auditory stimuli appears as positive deflections in newborns (e.g., Dehaene-Lambertz, 2000; Friederici et al., 2002; Winkler et al., 2003; Leppänen et al., 2004; Ruusuvirta et al., 2009; Cheng et al., 2012). The infant brain undergoes rapid maturational development, including myelination, synaptogenesis, and axonal connectivity. It has been suggested that the gradual shift in the synaptic and dendritic formation from deep to superficial cortical structures may contribute to the immature polarity of the cortical electrical dipole (Kostović and Judas, 2002). In addition, the observed positive MMR may also be due to the immature myelination, which makes the responses to standard stimuli refractory while the non-refractory responses to deviant stimuli remains positive, leaving the difference between deviant and standard responses positive in term newborns (Winter et al., 1995; Leppänen et al., 2004).

Three cautions should be kept in mind when interpreting the current result. Although it is exciting that the emotional effects observed in this study support the evolutionary importance of threatening voices that might be processed automatically in sleeping newborns (Grandjean et al., 2005; Vuilleumier, 2005; Belin and Grosbras, 2010), the result does not necessarily signify a discrimination based on emotional content. In other words, the ability to discriminate between emotional voices does not indicate neonates could derive emotional information from these vocal expressions (Leppänen and Nelson, 2008). Another aspect that deserves attention is the fact that the terms “fear” and “anger” used in this study should be taken as emotional labels as evaluated by adults and does not imply that the same emotions were necessarily evoked in neonates when hearing these stimuli (Blasi et al., 2011). However, this issue seems to be a common problem when investigating the emotional process in neonates and young infants. Finally, it is suggested that the sensitivity to threat-related emotion at birth not only has a potential advantage in the social cognitive development, but may also give a source of vulnerability in the emotion regulation of neonates and young infants.

## Conclusion remarks

Humans extract from voices a wealth of socially-relevant information that constitutes a universal and non-linguistic mode of communication (Latinus and Belin, 2011). Furthermore, a priority to threat-related prosodies serves an evolutionarily adaptive purpose and helps us appropriately avoiding harmful stimuli (Vaish et al., 2008). To answer the question that whether the sensitivity to different threat-related emotions is a basic characteristic of auditory perception that is present at birth, this study investigated the neural correlates underlying automatic processing of emotional voices of fear and anger in neonates. We found that the neonatal MMR component could discriminate fearful vocal sounds from angry vocal sounds, and that this fear–anger MMR separation was only observed in the fronto-central scalp when neonates were in AS state. Although neonates' perceptual sensitivity is not likely associated with a rich conceptual understanding of the meaning of fearful and angry emotions, this special discrimination in early life may provide a foundation for later emotion and social cognition development (Leppänen and Nelson, 2012; Vrtička and Vuilleumier, 2012).

## Conflict of interest statement

The authors declare that the research was conducted in the absence of any commercial or financial relationships that could be construed as a potential conflict of interest.
