# Supplementary material for: Discrimination of fearful and angry emotional voices in sleeping human neonates: a study of the mismatch brain responses
Source: Front Behav Neurosci. 2014 Dec 4;8:422. doi: 10.3389/fnbeh.2014.00422 (PMC4255595; doi:10.3389/fnbeh.2014.00422)
Supplement: Supplementary file 1 [file DataSheet1.DOCX]

Additional experiment

## Materials and methods

In the additional experiment, another 25 full-term neonates (13 boys; gestational age = 37 to 40 weeks) with postnatal ages that ranged from 0 to 6 days were included using the same inclusion/exclusion criteria as in the main experiment. The angry "dada" and fearful "dada" voices were randomly presented with equal probability (50% versus 50%) during the experiment. The EEG recording lasted for approximately 3.5 min and consisted of 100 angry and 100 fearful vocal trials. The interstimulus interval varied randomly between 550 and 750 ms. Based on the result of the main experiment, the additional experiment only recorded the EEG data at the electrode site of FC4 and during the AS stage. The ERP component was measured as the mean amplitudes within a time window of 300-500 ms post stimulus.

## Results

The ANOVA revealed that the two-way interaction of emotion by sound type was significant (*F*(1, 24) = 4.45, *p* = .046, $\eta_{p}^{2}$ = 0.156) (Figure S1). Simple effect analysis revealed that the ERP evoked by angry “dada” sounds (4.61 ± 1.24 μV) was significantly larger than that evoked by fearful “dada” sounds (3.51 ± 1.36 μV) in the vocal condition (*F*(1, 24) = 11.1, *p* = .003). However, this effect was not significant in the nonvocal condition (*F*(1, 24) < 1).

In addition, the main effect of sound type was significant (*F*(1, 24) = 104, *p* < .001, $\eta_{p}^{2}$ = 0.812). The ERP evoked by vocal sounds (4.06 ± 1.40 μV) was significantly larger than that evoked by nonvocal sounds (1.29 ± 1.26 μV).


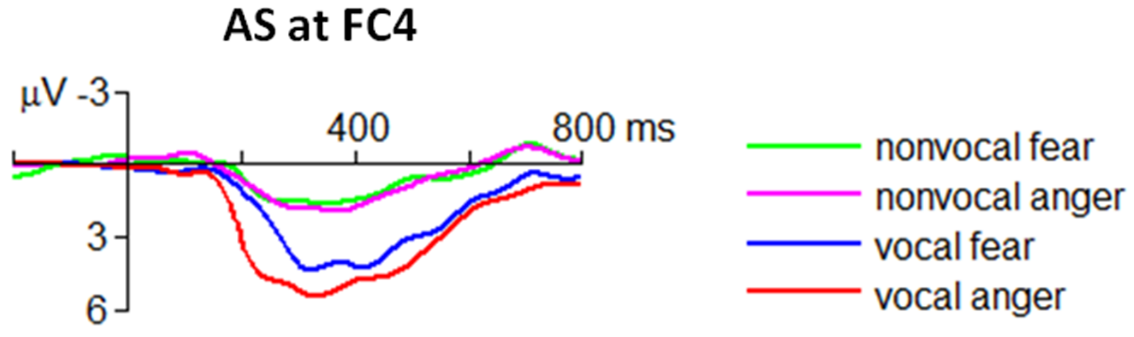


Figure S1. Grand-mean ERP waveforms in the four experimental conditions.

## Discussion

The result of the additional experiment revealed that even with equal presentation probability, neonates can still differentiate angry from fearful voices. The reason why anger elicited a more pronounced neural response than fear remains unclear. We suggest one of the reasons may be the need to understand/express negative intentions in interpersonal interactions, which are usually delivered by anger rather than fear (e.g., anger rather than fear is typically aroused when people are encountering interpersonal rejection) (Ackerman, et al., 2006; Leary, Twenge, & Quinlivan, 2006; Moons, Eisenberger, & Taylor, 2010). In our opinion, the sensitivity to anger provides neonates a cognitive foundation for efficient negative interactions with their parents, e.g., cry or anger often discourages future separation from parents while the emotion of fear would be less impressive (Bowlby, 1982).

## References

Ackerman, J. M., Shapiro, J. R., Neuberg, S. L., Kenrick, D. T., Becker, D. V., Griskevicius, V., Maner, J. K., & Schaller, M. (2006). They All Look the Same to Me (Unless They're Angry) From Out-Group Homogeneity to Out-Group Heterogeneity. *Psychological science, 17*, 836-840.

Bowlby, J. (1982). Attachment and loss: retrospect and prospect. *American Journal of Orthopsychiatry, 52*, 664.

Leary, M. R., Twenge, J. M., & Quinlivan, E. (2006). Interpersonal rejection as a determinant of anger and aggression. *Personality and Social Psychology Review, 10*, 111-132.

Moons, W.G., Eisenberger, N.I., & Taylor, S.E. (2010). Anger and fear responses to stress have different biological profiles. *Brain, Behavior, and Immunity*, 24, 215-219.
